# The psychosexual and psychosocial impacts of polygamous marriages: a cross-sectional study among Somali women

**DOI:** 10.1186/s12905-023-02830-1

**Published:** 2023-12-13

**Authors:** Adil Barut, Samira Ahmed Mohamud

**Affiliations:** Obstetrics and Gynaecology Department, Somali-Mogadishu Recep Tayyip Erdoğan Research and Training Hospital, Mogadishu, Somalia

**Keywords:** Polygamous marriages incidence, Somalia, Psychosocial, Psychosexual

## Abstract

**Background:**

Polygamous marriages are common in many Africa countries. This study aimed to document psychosexual and psychosocial problems of Somali women engaged in monogamous or polygamous marriages.

**Methods:**

This cross-sectional study included 607 consecutive women who had presented between June 7 and October 1, 2022, to the Department of Gynaecology of Mogadishu Somali Turkey Training and Research Hospital in Mogadishu, the capital city of Somalia. Data included maternal age, type of marriage (polygamy, monogamy, and arranged marriage), wives’ education, husbands’ education, husband income, residence area (rural or urban), number of marriages, living in houses (same or different), number of co-wives, and age of marriage. The participants were asked to complete three questionnaires: The Female Sexual Function Index (FSFI), the Rosenberg Self-Esteem Scale (RSE), and the Brief Symptom Inventory-18 (BSI-18).

**Results:**

Of 607 women, 435 (71.7%) had monogamous marriages and 172 (28.3%) had polygamous marriages. The mean age was 29.0 ± 7.2 years (range 16–46). In polygamous marriages, the mean number of wives a husband had was 2.4 ± 0.7 women (range 2– 4). The overall incidences of sexual dysfunction, low self-esteem and arranged marriage were 59.8%, 79.4% and 64.4%, respectively. Wives in polygamous marriages differed from those in monogamous marriages with significantly higher rate of illiterateness (41.9% vs. 27.4%, *p* = 0.004). Increases in husband income corresponded to higher rates of polygamous marriage. Women in polygamous marriages had significantly lower scores in the desire, arousal, orgasm, and satisfaction sub-domains. Sexual dysfunction, with a significantly increased rate among women in polygamous marriages. Polygamous marriages were associated with significantly higher levels of anxiety, and depression, and a significantly higher total BSI score (*p* = 0.010, *p* = 0.004, and *p* = 0.020, respectively). Women in both groups had similar levels of low self-esteem (*p* > 0.05). In univariate analysis, polygamous marriage was in significant inverse associations with the total FSFI score and subdomain scores of desire, arousal, orgasm, satisfaction, and sexual dysfunction and in significant associations with the BSI total score and subdomain scores of anxiety and depression (*p* < 0.05).

**Conclusion:**

Our findings suggest that women in polygamous marriages experience considerably higher psychosexual and psychosocial adverse effects as compared with their monogamous counterparts.

## Background

Polygamy is defined as a marital relationship involving multiple spouses. The most common form is polygyny, involving one husband who is married to two or more wives ADDIN EN.CITE [1, 2]. The prevalence of polygamous marriages across sub-Saharan African countries has been reported as 40% in the Republic of Chad, 37.9% in Benin, 37.4% in Mali, and 29.9% in Nigeria [3]. To our knowledge, there has been no estimate about the prevalence of polygamous marriages in Somalia.

Sexual function may differ in women engaged in monogamous and polygamous marriages because there are diverse psychological factors affecting women in the latter form of marriage. Sexual function is a component of health dynamically affected not only by individual characteristics such as age, psychosexual development, and sexual experiences but also by social values, social status, education, religion, sex-specific roles and expectations [4, 5]. In polygamous marriages, the roles of women show significant alterations because of varying social values and status in the family, rendering them more susceptible to sexual dysfunction that may manifest as decreased levels of desire, arousal, orgasm, and satisfaction. Possible adverse effects of polygamous marriages on sexual function have not received attention in the medical literature.

On the other hand, the psychological and emotional adverse effects of polygamous marriages have been documented in individual studies and meta-analyses. Women in polygamous marriages have been found to have lower levels of self-esteem and higher levels of depression and anxiety than their counterparts in monogamous marriages [6].

In Somalia, neither psychosexual nor psychosocial dimensions of polygamous marriages have been examined, particularly in comparison with monogamous marriages. This study aimed to document the psychosexual and psychosocial problems of Somali women engaged in monogamous or polygamous marriages who presented to the gynaecology clinic of the foremost hospital in the country. In a relatively large sample (n = 607), psychosexual status was evaluated using the Female Sexual Function Index (FSFI) and psychosocial status was evaluated using the Rosenberg Self-Esteem Scale (RSE) and the Brief Symptom Inventory-18 (BSI-18).

## Materials and methods

### Study design and participants

This cross-sectional study included data on 607 consecutive women who had presented between June 7 and October 1, 2022, to the Department of Gynaecology of Mogadishu Somali Turkey Training and Research Hospital in Mogadishu, the capital city of Somalia. Data included maternal age, type of marriage (polygamy, monogamy, and arranged marriage), wives’ education, husbands’ education, husband income, residence area (rural or urban), number of marriages, living in houses (same or different), number of co-wives, and age of marriage.

The participants were asked to complete three questionnaires: The Female Sexual Function Index (FSFI), the Rosenberg Self-Esteem Scale (RSE), and the Brief Symptom Inventory-18 (BSI-18). For those who were illiterate, the questionnaires were completed by a gynaecology specialist (AB or SM), consistent with responses given by the participants.

Inclusion criteria were being married (monogamy or polygamy) and sexually active, absence of comorbid diseases (e.g., renal or liver failure, diabetes mellitus, hypertension, and/or coronary artery disease), no history of a previous major pelvic trauma, psychiatric or neurological disorder, alcoholism, illicit drug use, and use of drugs that might affect sexual function.

### Questionnaires

The FSFI was developed by Rosen and colleagues (2000) to assess six domains of female sexual function (sexual desire, sexual arousal, lubrication, orgasm, satisfaction, and pain) and has become one of the most widely used measures of sexual functioning of women. The 19-item FSFI is easy to understand and has been adapted to a number of languages. The items are scored on a five-point (1 to 5) Likert scale, with lower scores corresponding to lower levels of sexual functioning and a total score of less than 26.55 indicating sexual dysfunction. Fifteen items also include a sixth response option scored with zero indicating no sexual activity in the past four weeks. Each individual domain score is calculated by the summation of individual scores of the domain multiplied by a factor for each domain ranging from 0.3 to 0.6. Finally, the individual domain scores are added to obtain the overall score [7, 8]. In reliability analysis, interrater reliability Cronbach’s alpha coefficient of the FSFI in the Somalian language was found as 0.88 (range 0.82–0.92), with a content-related validity coefficient of 0.35.

The BSI-18 is a short form of the Symptom Checklist-90-Revised. It contains the three six-item domains (somatization, depression, and anxiety), whose total score ranges from 0 to 72, with higher scores indicating increased negative feelings about the self [9, 10]. The interrater reliability Cronbach’s alpha coefficient and content-related validity coefficient of the BSI-18 in the Somalian language were 0.94 (range 0.86–0.96) and 0.32, respectively.

The RSE is a measure of self-esteem, consisting of 10 items concerning global self-worth inquiring into both positive and negative feelings about the self. All items are answered on a 4-point (1 to 4) Likert scale ranging from strongly agree to strongly disagree. A cut-off value of 15 distinguishes between normal and low self-esteem, with a lower score indicating the latter [11, 12]. In reliability analysis, the interrater reliability Cronbach’s alpha coefficient of the RSE in the Somalian language was found as 0.40, with a content-related validity coefficient 0.22.

### Ethics approval and consent to participate

The study was approved by the Ethics and Research Committee of Mogadishu Somali Turkey Training and Research Hospital (Permission number: MSTH/10,586/06.06.2022/613). The study was performed in accordance with the principles and guidelines of the Declaration of Helsinki. All participants were informed about the study and gave informed consent to publication of the results. For one participant who was younger than 16 years of age, informed consent was obtained from her parents. As a considerable proportion of the participants were illiterate, informed consent was obtained from their legal representatives. Analysis and reporting of the results are in compliance with the Strengthening the Reporting of Observational Studies in Epidemiology (STROBE) checklist.

### Definitions

Arranged marriage is defined as a marital union where the two spouses are primarily united by individuals other than the couple themselves, particularly by family members.

### Data processing and analysis

Since there has been no previous study assessing sexual function in polygamous marriages, a power analysis was performed using the G*Power program and a sample size of 164 participants for monogamous and polygamous groups each was found to be sufficient for statistical analysis. Data were collected using a structured format including relevant sociodemographic features and were processed using the Statistical Package for Social Sciences (SPSS) version 21 (IBM Corp., Armonk, N.Y.; USA). Quantitative data were expressed as means, standard deviation (SD), median, minimum, and maximum, and qualitative data as frequencies and percentages. Homogeneity was checked using Levene’s test, where a p-value of > 0.05 was considered in favour of homogeneity. The Shapiro-Wilk normality test was used to check whether continuous variables were normally distributed.

For pairwise comparisons, numerical variables were compared using the independent t-test if normally distributed. Multigroup comparisons of normally distributed variables were made using the one-way ANOVA test. The Post Hoc Multiple Comparisons (Bonferroni) were used to determine between-group differences. Nominal variables were analysed with Pearson’s or Fisher’s chi-squared test. Regression analysis was performed to determine the effect of polygamy on the parameters of psychosocial and psychosexual function. A p value of less than 0.05 was accepted as statistically significant. All variables were expressed with 95% confidence intervals (CI).

## Results

During the study period, a total of 2405 women presented to the gynaecology outpatient clinic. Due to physical limitations and the time required for the completion of each questionnaire, a random selection was made, pointing to the third presenting patient after the completion of each set of questionnaires. As a result, 922 women were asked to participate in the study, and 607 women gave consent and completed the three questionnaires. The questionnaires of 191 illiterate women (31.5%) were completed by the investigators in compliance with the responses obtained. Each set of questionnaires took approximately 30 min to complete for literate women and 40 min for illiterate women.

### Sociodemographic characteristics

Of 607 participating women, 435 had monogamous marriages, and 172 had polygamous marriages. The mean age was 29.0 ± 7.2 years (range 16–46). The mean age at marriage was 20.0 ± 3.9 years (range 10–34). The characteristics of the participants are summarised in Table 1.

**Table 1:** Sociodemographic, psychosexual and psychosocial characteristics of Somali married women

	Mean ± SD	Count	Percent
Age of marriage (years)	20.0 ± 3.9		
Current age	29.0 ± 6.9		
Type of marriage			
Monogamy		435	71.7
Polygamy		172	28.3
Two co-wives		114	66.3
Three co-wives		41	23.8
Four co-wives		17	9.9
Co-wives			
First wife		68	39.5
Second co-wife		82	47.7
Third co-wife		17	9.9
Fourth co-wife		5	2.9
Residence			
Rural		538	88.6
Urban		69	11.4
Education status			
Illiterate		191	31.5
Primary school		99	16.3
Secondary and/or high school		189	31.1
University		127	21.1
Education status of the husband			
Illiterate		147	24.2
Primary school		166	27.3
Secondary and/or high school		16	2.6
University		278	45.8
Family income ($ USA)			
< 500		290	47.8
500–1000		296	48.8
> 1000		21	3.4
Arranged marriage		391	64.4
Sexual dysfunction **		363	59.8
RSE score*			
Low self-esteem		482	79.4
Normal		125	20.6

*A RSE score of < 15 indicates low self-esteem. **A FSFI score of < 26.55 indicates sexual dysfunction

### Incidences of polygyny, sexual dysfunction and arranged marriages

In polygamous marriages, the mean number of wives a husband had was 2.4 ± 0.7 women (range 2– 4). Of 607 women, 172 (28.3%) had polygamous marriages. The majority of polygamous marriages (66.3%, 114/172) consisted of two wives, followed by three wives (23.8%, 41/172) and four wives (9.9%, 17/172) forms. The incidences of sexual dysfunction, low self-esteem and arranged marriage were 59.8%, 79.4% and 64.4%, respectively (Table 1).

### Between-group comparisons

Wives in polygamous marriages differed from those in monogamous marriages with a significantly higher rate of illiterateness (41.9% vs. 27.4%, *p* = 0.004) and a lower rate of university education (14% vs. 23.9%, *p* = 0.004).

In addition, increases in husband income in USA dollars corresponded to higher rates of polygamous marriage, being 37.2% with a lower income of < 500 $ USA, 57% with an income of 500–1000 $ USA, and 5.8% with an income of > 1000 $ USA, as compared with 52%, 45.5%, and 2.5% in the monogamy group, respectively (p = 0.002). The two groups were similar with respect to the age at marriage, education status of the husbands, rate of arranged marriages and area of residency (*p* > 0.05) (Table 2).

**Table 2:** Comparison of polygamy and monogamy marriages with respect to education status, area of residency, arranged marriage, husband income, scores on FSFI, BSI, and RSE

Parameters	Monogamy (n = 435), n (%)	Polygamy (n = 172), n (%)	p*
Married age (years), mean ± SD	19.9 ± 3.7	20.2 ± 4.4	0.468*
Education status (wives) n (%)			0.004**
Illiterate	119 (27.4)	72 (41.9)	
Primary school	73 (16.8)	26 (15.1)	
Secondary and/or high school	139 (32.0)	50 (29.0)	
University	104 (23.9)	24 (14)	
Education status (husbands) n (%)			0.085**
Illiterate	94 (21.6)	53 (30.8)	
Primary school	122 (28)	44 (25.6)	
Secondary and/or high school	10 (2.3)	6 (3.5)	
University	209 (48)	69 (40.1)	
Area of residency			0.660**
Rural	384 (88.3)	154 (89.5)	
Urban	51 (11.7)	18 (10.5)	
Arranged marriage n (%)	278 (63.9)	113 (65.7)	0.678**
Sharing the same house n (%)	NA	20 (11.6)	NA
Living in different houses n (%)	NA	152 (88.4)	
Husband income ($ USA) n (%)			0.002**
< 500	226 (52.0)	64 (37.2)	
500–1000	198 (45.5)	98 (57.0)	
> 1000	11 (2.5)	10 (5.8)	
FSFI mean ± SD			
Total score	25.1 ± 5.0	23.7 ± 5.1	0.002*
Desire	4.4 ± 1.3	4.0 ± 1.3	0.002*
Arousal	4.5 ± 1.2	4.0 ± 1.2	0.0001*
Lubrication	4.0 ± 1.1	3.9 ± 1.1	0.161*
Orgasm	4.4 ± 1.1	4.1 ± 1.1	0.006*
Satisfaction	4.6 ± 1.2	4.3 ± 1.3	0.0001*
Pain	3.3 ± 0.9	3.4 ± 0.8	0.466*
Sexual dysfunction n (%)	243 (55.9)	120 (69.8)	0.002*
BSI mean ± SD			
Total score	12.0 ± 11.2	14.4 ± 10.7	0.020*
Anxiety	4.04 ± 4.4	5.1 ± 4.2	0.010*
Somatization	4.63 ± 4.4	4.9 ± 4.4	0.428*
RSE n (%)			
Low self-esteem	347 (79.8)	135 (78.5)	0.725*

**Chi-squared test; *Independent-Samples T Test; NA: Not applicable; SD: standard deviation; FSFI: Female Sexual Function Index; BSI: Brief Symptom Inventory; RSE: Rosenberg Self-Esteem.

Comparisons of the two groups with respect to the scores on FSFI, BSI, and RSE are presented in Table 2. Although women in monogamous marriages had significantly higher scores in the desire, arousal, orgasm, and satisfaction sub-domains and a significantly higher total score, sexual dysfunction, as defined by a total score of less than 26.55, was prevalent in both groups (55.9% for monogamous vs. 69.8% for polygamous marriages), with a significantly increased rate among women in polygamous marriages (*p* = 0.002).

Women in polygamous marriages had significantly higher BSI scores except for the somatization score. Monogamous marriages were associated with significantly lower levels of anxiety and depression, and a significantly lower total BSI score (*p* = 0.010, *p* = 0.004, and *p* = 0.020, respectively). Based on the RSE scores, women in both groups had similar levels of low self-esteem (p > 0.05).

Second co-wives significantly differed from women in the monogamy group with respect to decreased mean levels of desire (3.9 ± 1.2 vs. 4.4 ± 1.3, *p* = 0.006), arousal (3.9 ± 1.2 vs. 4.5 ± 1.2, *p* = 0.002), and satisfaction (4.1 ± 1.25 vs. 4.6 ± 1.2, *p* = 0.004) (Table 3). Similarly, co-wives following the second co-wives had significantly lower mean scores than the monogamy group for desire (3.8 ± 1.2 vs. 4.4 ± 1.3, p = 0.009) and arousal (3.6 ± 1.2 vs. 4.5 ± 1.2, *p* = 0.018). Conversely, first co-wives had significantly increased mean levels of anxiety and depression as compared with the monogamy group and other co-wives (Table 4).

**Table 3:** Between-group comparisons of monogamous and polygamous wives with respect to scores on FSFI and BSI

Parameters	Monogamy (n = 435) Mean ± SD	First wife (n = 68) Mean ± SD	Second Co-wife (n = 82) Mean ± SD	Third-fourth co-wife (n = 22) Mean ± SD	p*
FSFI					
Desire	4.4 ± 1.3	4.3 ± 1.3	3.9 ± 1.2	3.8 ± 1.2	**P2:0.006** **P3:0.009**
Arousal	4.5 ± 1.2	4.3 ± 1.2	3.9 ± 1.2	3.6 ± 1.3	**P2:0.002** **P3:0.018**
Lubrication	4.0 ± 1.1	3.9 ± 1.0	3.9 ± 1.1	3.5 ± 1.1	NS
Orgasm	4.4 ± 1.1	4.2 ± 1.0	4.1 ± 1.1	3.8 ± 1.1	NS
Satisfaction	4.6 ± 1.2	4.5 ± 1.2	4.1 ± 1.3	3.9 ± 1.4	**P2:0.004** **P3:0.049**
Pain	3.3 ± 0.9	3.2 ± 0.8	3.5 ± 0.8	3.4 ± 0.9	NS
BSI					
Anxiety	4.0 ± 4.4	6.7 ± 4.4	4.1 ± 4.0	3.6 ± 2.9	**P1:0.0001** **P2:0.002** **P3:0.025**
Somatization	4.6 ± 4.4	4.8 ± 4.1	5.0 ± 4.7	5.1 ± 4.4	NS
Depression	3.3 ± 3.7	5.0 ± 4.8	3.4 ± 3.7	2.7 ± 2.2	**P1:0.0001** **P2:0.0001** **P3:0.003**

NS: Not significant; One-Way ANOVA Test (Bonferroni); SD: standard deviation; FSFI: Female Sexual Function Index; BSI: Brief Symptom Inventory; *P1: Monogamy- first wife; p2: Monogamy- second co-wife; p3: Monogamy-third-forth co-wife

**Table 4:** Regression analysis showing associations between polygamous marriage and FSFI, BSI and RSE scores

Multivariate analysis		OR	95% CI		p
Education status (wives)					
Illiterate		2.62	1.54	4.46	**0.001**
Primary school		1.28	0.99	1.65	0.060
Secondary and/or high school		1.50	0.86	2.61	0.154
Education status (husbands)					
Illiterate		1.71	1.11	2.63	**0.015**
Primary school		1.11	0.70	1.69	0.693
Secondary and/or high school		1.01	0.20	5.13	0.988
Husband income ($ USA)					
500–1000		1.75	1.21	2.53	**0.003**
> 1000		3.21	1.30	7.90	**0.011**
**Binary analysis**					
Area of residency		0.88	0.50	1.55	0.660
Arranged marriage		1.08	0.75	1.57	0.678
RSE low self-esteem		0.93	0.60	1.43	0.725
Sexual dysfunction		1.82	1.25	2.66	**0.002**
**Univariate analysis**	**Estimate**	**SE**	**95% CI**		
FSFI					
FSFI total score	-1.39	0.453	-2.28	-0.503	**0.002**
Desire	-0.355	0.1138	-0.578	-0.131	**0.002**
Arousal	-0.428	0.1099	-0.644	-0.213	**0.0001**
Lubrication	-0.139	0.0987	-0.332	0.0553	0.161
Orgasm	-0.267	0.0969	-0.458	-0.0772	**0.006**
Satisfaction	-0.393	0.1122	-0.613	-0.172	**0.0001**
Pain	0.0574	0.0787	-0.0972	0.212	0.466
BSI					
BSI total score	2.34	1.00	0.372	4.30	**0.02**
Anxiety	1.02	0.392	0.25	1.79	**0.01**
Somatization	0.314	0.396	-0.463	1.09	0.428
Depression	1.00	0.346	0.322	1.68	**0.004**

Reference: Monogamous marriage for sexual function, university education for education level, <$500 for income. FSFI: Female Sexual Function Index; BSI: Brief Symptom Inventory;

RSE: Rosenberg Self-Esteem. OR: Odds ratio; SE: Standard error, CI: Confidence interval

### Regression analysis

The results of the regression analysis are summarised in Table 4. Illiteracy of both wives and husbands and husband income level showed significant associations with polygamous marriage. Polygamous marriage was in significant inverse associations with the total FSFI score and subdomain scores of desire, arousal, orgasm, satisfaction, and sexual dysfunction and in significant associations with the BSI total score and subdomain scores of anxiety and depression (*p* < 0.05) (Table 4).

## Discussion

We evaluated the impact of polygamous marriage on psychosexual and psychosocial dimensions as determined by the FSFI, BSI and RSE scores in Somali women. To our knowledge, this is the first study in Somalia and Africa involving one of the largest series of monogamous and polygamous marriages to examine the psychosexual and psychosocial impacts of polygamous marriages. The prevalence of polygamous marriages in our sample was remarkably high at 28.3%, representing the fifth highest reported rate across all sub-Saharan Africa countries, following the Republic of Chad (40%), Benin (37.9%), Mali (37.4%), and Nigeria (29.9%) [3].

In this study, the majority of polygamous marriages included two co-wives (66.3%), followed by three co-wives (23.8%) and four co-wives (9.9%). This is also the first study to examine individual psychosexual and psychosocial impacts of polygamous marriages on each co-wife in marital integrity.

In this study, women in polygamous marriages had significantly lower FSFI scores (total score, desire, arousal, orgasm, and satisfaction) and significantly higher BSI scores (total score, anxiety, and depression). These findings indicate the detrimental psychosexual and psychosocial effects of polygamous marriages when compared with women in monogamous marriages. Reports on both psychosexual and psychosocial impacts of polygamous marriages are lacking because psychosexual impacts on polygamous women have not received sufficient attention in the medical literature. A systematic review and meta-analysis that included 18 studies from ten countries assessed the psychological impacts of polygamous marriages on women and reported a significantly higher odds ratio for depression. However, polygamous marriages were not associated with psychological distress and anxiety [1]. In another systematic review and meta-analysis that included ten studies from different countries (Palestine, Turkey, Iran, Syria, Israel, Tanzania and Jordan), women in polygamous marriages were reported to have low self-esteem, somatic symptom disorder, depression, and anxiety [13]. In a similar study, polygamous marriages were associated with psychological disorders such as somatic symptom disorder, depression and anxiety [14].

Our findings also showed a significantly increased incidence of sexual dysfunction, defined as a total score of less than 26.55, among women in polygamous marriages (69.8%) as compared with monogamous peers (55.9%). A similar comparison between polygamous and monogamous women with respect to the incidence of sexual dysfunction could not be found in the literature.

Our findings also support a previously reported phenomenon of the first-wife syndrome [15, 16] among polygamous women, characterised by the highest levels of anxiety and depression in the first wife as compared with other co-wives in a polygamous marriage (Table 3). Understandably enough, the first co-wife would be unwilling to share her husband within the same house or in another house and may response with emotional upset or anger, negative attitudes towards her husband as well as hostility towards the other wife/wives.

Apart from psychosexual and psychosocial detrimental effects of polygamous marriages on women, our findings indicate three interesting features and rationale of polygamous marriages. First, at all education levels (primary, secondary, high), the proportions of polygamous women were lower as compared with monogamous counterparts. This difference was particularly striking and significant among illiterate women (41.9% for polygamous vs. 27.4% for monogamous women), suggesting that polygamous marriages might find considerable support from illiteracy. Second, the proportions of polygamous women showed an inverse relationship with the level of husband income. The higher income the husband had, the higher proportion of polygamous women as compared with monogamous women having the same income status. Third, only 11.6% of polygamous women reported living in the same house with their co-wives, while 88.4% lived apart from the other co-wives.

### Limitations

Although our study provides clear-cut data with a considerably large sample size about the current condition of sexual function, psychological and emotional distress among Somali women in polygamous marriages, it reflects the single-centre experience. It thus may not be representative of the general population.

## Conclusion

Our findings suggest that women in polygamous marriages experience considerably higher psychosexual and psychosocial adverse effects as compared with their monogamous counterparts. This study also shows that, in underdeveloped countries like Somalia, illiterateness of women constitutes one of the major contributors to polygamous marriages.

## Data Availability

All data generated or analysed during this study are included in this article. The datasets used and/or analysed during the current study are available from the corresponding author on reasonable request, but restrictions apply to the availability of these data, which were used under license for the current study, and so are not publicly available. The corresponding author (email: dradilbarut@gmail.com) can be contacted for the data with a reasonable request.

## Abbreviations

OR: Odds ratio
CI: Confidence interval
OR: Odds ratio
SPSS: Statistical package for social science
SD: Standard deviation
STROBE: Strengthening the Reporting of Observational Studies in Epidemiology
FSFI: Female Sexual Function Index
RSE: Rosenberg Self-Esteem Scale
BSI-18: Brief Symptom Inventory-18
$: Dollar
USA: United States of America
IBM: International Business Machines
